# Observations on Hydrophobia

**Published:** 1811-09

**Authors:** 


*To the Editors of the Medical and Physical Journal.*

Gentlemen,

If you think the following facts worthy of being recorded, I beg you will insert them in your valuable Journal. The veracity of these facts you may depend upon. The gentleman, at whose country residence they happened, is still living in the neighbourhood of the metropolis.

Some years back a dog, in a state of hydrophobia, made his way into a farm-yard belonging to the above gentleman, and there bit a dog, some swine, and a cow; the animals were immediately confined. In about five or six weeks the dog died rabid, the swine in the same state also. The cow, which was known to have been bitten, was, soon after the death of these animals, taken very ill with the like symptoms; it was supposed she must very soon have died had no means of recovery been resorted to. The gentleman had at the time a bottle of antimonial wine in the house, he designed to try the effect of this medicine on the animal, and poured the contents of the whole bottle down the cow's throat. After some time the cow broke out into a most violent perspiration, so much so that the sweat ran off her profusely; the expression made use of when I was made acquainted with the fact was, that the perspiration lay in lakes between the hollow parts of the cow's back. In a short time after this perspiration the cow grew better, and at last perfectly recovered.

I should think from the account given, the complaint was a confirmed case of hydrophobia; but whether this medicine has ever been given in this most dreadful disease, I know not; or if it has been given in sufficient quantity.

I remain,

Your most obedient Servant,

VERITAS.*

*Liverpool, July* 12, 1811.

---

* The Editors generally decline the insertion of extraordinary occurrences upon anonymous authority. For once they have ventured to depart part from their established principle of conduct, with the hope that the gentleman, at whose country residence the case occurred, will confirm the fact. Perhaps accident may some time discover a remedy for this dreadful malady, when theory, science, and reasoning, have failed.

